# Bis(tetra­butyl­ammonium) bis­(3,4,5-trioxocyclo­pent-1-ene-1,2-dithiol­ato-κ^2^ *S*,*S*′)cadmate(II) 0.25-hydrate

**DOI:** 10.1107/S1600536810049913

**Published:** 2010-12-04

**Authors:** Hong-Yu Chen, Guang-Ming Xia, Zhen-Wei Zhang, Ping Li

**Affiliations:** a School of Chemistry and Chemical Engineering, TaiShan Medical University, Tai’an 271016, People’s Republic of China; b Shandong Provincial Key Laboratory of Fluorine Chemistry and Chemical Materials, School of Chemistry and Chemical Engineering, University of Jinan, Ji’nan 250022, People’s Republic of China

## Abstract

The title compound, (C_16_H_36_N)_2_[Cd(C_5_O_3_S_2_)_2_]·0.25H_2_O, contains two disordered tetra­butyl­ammonium cations, a complex [Cd(C_5_O_3_S_2_)_2_]^2−^ anion and a 0.25-hydrate water. The anion is composed of a bidentate coordinated 3,4,5-trioxocyclo­pent-1-ene-1,2-dithiol­ate (dtcroc) group forming a distorted tetra­hedral configuration around the Cd^II^ ion. The dihedral angle between the least-squares planes of the ten-atom sulfur-substituted croconate groups in the anion is 84.10 (8)°. The crystal packing is stabilized by weak C—H⋯O and C—H⋯S cation–anion hydrogen-bond inter­actions. In each of the two cations one butyl group is disordered over  two positions in the ratios 0.589 (11):0.411 (11) and 0.796 (12):0.204 (12).

## Related literature

For the delocalized electronic structures, redox chemistry and range of coordination geometries of metal complexes of chelating ethyl­ene-1,2-dithiol­ato ligands, see: Eisenberg (1970 ▶); Kato (2004 ▶). For the coordination behavior of the dtcroc dianion, see: Deplano *et al.* (2005 ▶, 2006 ▶). For related structures, see: Dunitz *et al.* (2001 ▶); Castro *et al.* (2002 ▶); Maji *et al.* (2004 ▶).
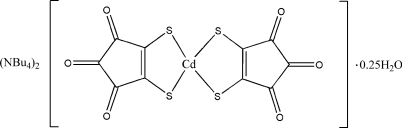


## Experimental

### 

#### Crystal data

(C_16_H_36_N)_2_[Cd(C_5_O_3_S_2_)_2_]·0.25H_2_O
*M* *_r_* = 946.16
Triclinic, 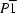

*a* = 9.820 (5) Å
*b* = 15.002 (5) Å
*c* = 17.406 (5) Å
α = 74.853 (5)°
β = 86.898 (5)°
γ = 87.705 (5)°
*V* = 2470.6 (17) Å^3^
*Z* = 2
Mo *K*α radiation
μ = 0.65 mm^−1^
*T* = 293 K
0.24 × 0.18 × 0.15 mm

#### Data collection

Oxford Diffraction CCD area-detector diffractometer
Absorption correction: multi-scan (*CrysAlis RED*; Oxford Diffraction, 2007 ▶) *T*_min_ = 0.775, *T*_max_ = 0.858
30150 measured reflections
10069 independent reflections
6510 reflections with *I* > 2σ(*I*)
*R*_int_ = 0.032

#### Refinement

*R*[*F* ^2^ > 2σ(*F* ^2^)] = 0.050
*wR*(*F* ^2^) = 0.144
*S* = 1.04
10069 reflections
534 parameters
54 restraints
H-atom parameters constrained
Δρ_max_ = 0.72 e Å^−3^
Δρ_min_ = −0.42 e Å^−3^

### 

Data collection: *CrysAlis PRO* (Oxford Diffraction, 2007 ▶); cell refinement: *CrysAlis PRO*; data reduction: *CrysAlis RED* (Oxford Diffraction, 2007 ▶); program(s) used to solve structure: *SIR97* (Altomare *et al.*, 1999 ▶); program(s) used to refine structure: *SHELXL97* (Sheldrick, 2008 ▶); molecular graphics: *SHELXTL* (Sheldrick, 2008 ▶); software used to prepare material for publication: *WinGX* (Farrugia, 1999 ▶).

## Supplementary Material

Crystal structure: contains datablocks global, I. DOI: 10.1107/S1600536810049913/jj2066sup1.cif

Structure factors: contains datablocks I. DOI: 10.1107/S1600536810049913/jj2066Isup2.hkl

Additional supplementary materials:  crystallographic information; 3D view; checkCIF report

## Figures and Tables

**Table 1:** Hydrogen-bond geometry (Å, °)

*D*—H⋯*A*	*D*—H	H⋯*A*	*D*⋯*A*	*D*—H⋯*A*
C12—H12*A*⋯O6^i^	0.97	2.48	3.412 (6)	161
C14—H14*C*⋯S2^ii^	0.97	2.70	3.472 (10)	137
C16—H16*B*⋯O2^iii^	0.97	2.54	3.483 (7)	163

Symmetry codes: (i) 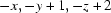; (ii) 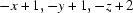; (iii) 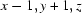.

**Fig. 1.**
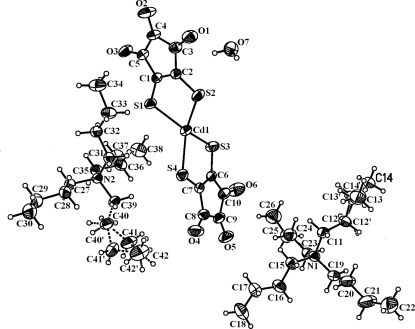
Coordination configuration of the title compound with thermal ellipsoids at 30% probability levels. Hydrogen atoms have been omiitted for clarity. Dashed lines indicate disordered butyl groups.

**Fig. 2.**
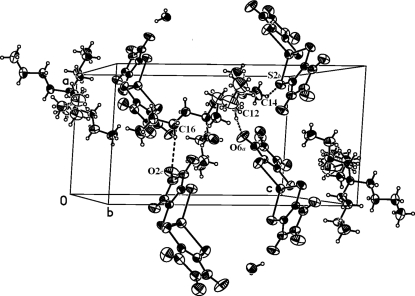
A packing diagram viewed down the b axis. symmetry code: a(-x, 1-y, 2-z); b(1-x, 1-y, 2-z); c(-1+x, 1+y, z);

## supplementary crystallographic information

### Comment

Metal complexes of chelating ethylene-1,2-dithiolato ligands (metal dithiolene)are of continuing interest owing to their delocalized electronic structures, rich redox chemistry and range of coordination geometries (Eisenberg,1970; Kato, 2004). They have also proven useful as precursors for the preparation ofnovel molecular conductors, non-linear optical materials and magnetic charge-transfer salts. Accordingly, we have interests in investigating the solid chemistry based on the 3,4,5-trioxo-cyclopent-1 ene-ene-1, 2-dithiolate ion (C_5_O_3_S_2_^2-^), also abbreviated as dtcroc (alternative name sulfur-substituted croconate). The coordination behavior of the dtcroc dianion (C_5_O_3_S_2_^2-^) towards d^8^ transition metal ions, such as Pt^2+^(Deplano,*et al.* 2005) and Ni^2+^(Deplano,*et al.* 2006), have been investigated and all show a planar configuration. We present here the synthesis and characterization of a new Cd(II) coordination compound of dtcroc, which shows a distorted tetrahedral coordination conformation.

The asymmetric unit of title compound, (C_16_H_36_N)_2_, [C_10_CdO_6_S_4_], 0.25(H_2_O) contains two disordered tetrabutylammonium cations, a coordinated [Cd(C_5_O_3_S_2_)_2_]^2-^ anion, and a 0.25 hydrate water (Fig. 1).  The [Cd(C_5_O_3_S_2_)_2_]^2-^ anion is composed of a bidentate coordinated dtcroc group forming a distorted tetrahedral configuration around a Cd (II) ion with Cd—S bonds between 2.5254 (13)–2.5413 (12)Å and six S—Cd—S angles between adjacent sulfur atoms in the coordination sphere close to 109.5°. The dihedral angle between the least-square-planes of the ten atom sulfur-substituted croconate group in the (C_5_O_3_S_2_^2-^) anion is 84.10 (8)°.

Deviations of oxygen atoms, sulfur atoms and the cyclic five-membered ring in C_5_O_3_S_2_^2-^ are less than 0.09 Å, indicating a planar molecular geometry for both ligands.  The C≐O bonds in the title compound, vary by 1.209 (5)–1.224 (5)Å and show typical C*sp*^2^ double bond character, while the C≐S bonds are in the range of 1.683 (4)–1.699 (4) Å, which are intermediate between the lengths of typical single C—S and double C≐S bonds.  The difference among C—C distances within the ligands are in the range of 1.402 (5)–1.505 (6) Å, establishing a π-electron localized C_2v_ molecular symmetry which compares well with similar structure croconate anions (Dunitz, *et al.*, 2001; Castro, *et al.*, 2002; Maji, *et al.*2004).

The tetrabutlyammionium cations are disordered in the title crystal and a few solvent water molecules were cocrystalized to stabilize the structure. The [Cd(C_5_O_3_S_2_)_2_]^2-^ units form stacks along [1 0 0], surrounded by tetrabutylamonium cations (Fig.2).  Crystal packing is stabilized by weak C—H···O, C—H···S cation–anion hydrogen bond interactions (Table 1).

### Experimental

To a solution containing K_2_dtcroc (0.2 g, 0.8 mmol) in H_2_O (20 mL) was addedto a solution containing Cd(NO_3_).4H_2_O (0.12 g, 0.4 mmol) in H_2_O (5 mL). The resulting mixture was heated to 70 °C for 1 h, then filtered into a solution of NBu_4_Br (0.40 g, 0.95 mmol) in ethanol (5 mL). Solid product was collected by suction filtration, washed with water and dried in air. Red block crystals were obtained by recrystallization from acetone.

### Refinement

All H atoms were geometrically fixed and allowed to ride on their attached atoms, which O—H = 0.85Å with *U*_iso_(H)= 1.5*U*_eq_(O) and C—H = 0.96–0.97Å with *U*_iso_(H)= 1.2–1.5*U*_eq_(C). Butyl groups are disordered in title structure. Some butyl groups were refined as a rigid body, which C—C bond are fixed to 1.54Å and the distances between alternate C atom are fixed to 2.54 Å. Terminal ethyl group C13—C14 is refined to a rigid model around the bond C11—C12 with ethyl group C13'-C14' in the ratio 0.59:0.41 and C41—C42 is refined to a rigid model around the bond C39—C40 with ethyl group C41'-C42' in the ratio 0.80:0.20.

### Crystal data

**Table:**

(C_16_H_36_N)_2_[Cd(C_5_O_3_S_2_)_2_]·0.25H_2_O	*Z* = 2
*M**_r_* = 946.16	*F*(000) = 1001
Triclinic, *P*1	*D*_x_ = 1.272 Mg m^−^^3^
Hall symbol: -P 1	Mo *K*α radiation, λ = 0.71069 Å
*a* = 9.820 (5) Å	Cell parameters from 6268 reflections
*b* = 15.002 (5) Å	θ = 2.5–27.5°
*c* = 17.406 (5) Å	µ = 0.65 mm^−^^1^
α = 74.853 (5)°	*T* = 293 K
β = 86.898 (5)°	Prism, red
γ = 87.705 (5)°	0.24 × 0.18 × 0.15 mm
*V* = 2470.6 (17) Å^3^	

### Data collection

**Table:**

Oxford Diffraction CCD area-detector diffractometer	10069 independent reflections
Radiation source: fine-focus sealed tube	6510 reflections with *I* > 2σ(*I*)
graphite	*R*_int_ = 0.032
φ and ω scans	θ_max_ = 26.4°, θ_min_ = 3.1°
Absorption correction: multi-scan (*CrysAlis RED*; Oxford Diffraction, 2007)	*h* = −12→12
*T*_min_ = 0.775, *T*_max_ = 0.858	*k* = −18→18
30150 measured reflections	*l* = −21→21

### Refinement

**Table:**

Refinement on *F*^2^	Primary atom site location: structure-invariant direct methods
Least-squares matrix: full	Secondary atom site location: difference Fourier map
*R*[*F*^2^ > 2σ(*F*^2^)] = 0.050	Hydrogen site location: inferred from neighbouring sites
*wR*(*F*^2^) = 0.144	H-atom parameters constrained
*S* = 1.04	*w* = 1/[σ^2^(*F*_o_^2^) + (0.0756*P*)^2^ + 0.4766*P*] where *P* = (*F*_o_^2^ + 2*F*_c_^2^)/3
10069 reflections	(Δ/σ)_max_ = 0.001
534 parameters	Δρ_max_ = 0.72 e Å^−^^3^
54 restraints	Δρ_min_ = −0.42 e Å^−^^3^

### Special details

**Table:**

Geometry. All e.s.d.'s (except the e.s.d. in the dihedral angle between two l.s. planes) are estimated using the full covariance matrix. The cell e.s.d.'s are taken into account individually in the estimation of e.s.d.'s in distances, angles and torsion angles; correlations between e.s.d.'s in cell parameters are only used when they are defined by crystal symmetry. An approximate (isotropic) treatment of cell e.s.d.'s is used for estimating e.s.d.'s involving l.s. planes.
Refinement. Refinement of *F*^2^ against ALL reflections. The weighted *R*-factor *wR* and goodness of fit *S* are based on *F*^2^, conventional *R*-factors *R* are based on *F*, with *F* set to zero for negative *F*^2^. The threshold expression of *F*^2^ > σ(*F*^2^) is used only for calculating *R*-factors(gt) *etc*. and is not relevant to the choice of reflections for refinement. *R*-factors based on *F*^2^ are statistically about twice as large as those based on *F*, and *R*- factors based on ALL data will be even larger.

### Fractional atomic coordinates and isotropic or equivalent isotropic displacement parameters (Å^2^)

**Table:**

	*x*	*y*	*z*	*U*_iso_*/*U*_eq_	Occ. (<1)
C1	0.5036 (3)	0.0335 (2)	0.73690 (19)	0.0507 (8)	
C2	0.6118 (4)	0.0659 (2)	0.7719 (2)	0.0542 (8)	
C3	0.7159 (4)	−0.0061 (3)	0.7956 (2)	0.0673 (10)	
C4	0.6686 (4)	−0.0890 (3)	0.7720 (3)	0.0670 (10)	
C5	0.5340 (4)	−0.0624 (3)	0.7358 (2)	0.0607 (9)	
C6	0.1825 (4)	0.3965 (2)	0.7705 (2)	0.0564 (9)	
C7	0.2513 (4)	0.4415 (2)	0.6991 (2)	0.0537 (8)	
C8	0.1950 (5)	0.5367 (3)	0.6716 (3)	0.0703 (11)	
C9	0.0873 (4)	0.5494 (3)	0.7309 (3)	0.0723 (11)	
C10	0.0816 (4)	0.4599 (3)	0.7949 (3)	0.0733 (11)	
C11	0.2345 (4)	0.7393 (3)	0.9656 (2)	0.0725 (11)	
H11A	0.2322	0.6810	0.9515	0.087*	
H11B	0.3240	0.7645	0.9489	0.087*	
C12	0.2188 (5)	0.7200 (3)	1.0553 (2)	0.0859 (13)	0.589 (11)
H12A	0.1385	0.6838	1.0749	0.103*	0.589 (11)
H12B	0.2086	0.7776	1.0709	0.103*	0.589 (11)
C13	0.3488 (11)	0.6657 (10)	1.0910 (6)	0.090 (5)	0.589 (11)
H13A	0.3774	0.6218	1.0608	0.108*	0.589 (11)
H13B	0.4222	0.7082	1.0872	0.108*	0.589 (11)
C14	0.3200 (11)	0.6145 (6)	1.1781 (5)	0.111 (4)	0.589 (11)
H14A	0.4001	0.5796	1.1988	0.167*	0.589 (11)
H14B	0.2461	0.5734	1.1818	0.167*	0.589 (11)
H14C	0.2958	0.6584	1.2083	0.167*	0.589 (11)
C12'	0.2188 (5)	0.7200 (3)	1.0553 (2)	0.0859 (13)	0.411 (11)
H12C	0.1266	0.7004	1.0723	0.103*	0.411 (11)
H12D	0.2320	0.7767	1.0706	0.103*	0.411 (11)
C13'	0.3202 (16)	0.6453 (13)	1.0989 (11)	0.098 (8)	0.411 (11)
H13C	0.3060	0.6354	1.1560	0.117*	0.411 (11)
H13D	0.3058	0.5874	1.0858	0.117*	0.411 (11)
C14'	0.4663 (15)	0.6772 (11)	1.0729 (10)	0.133 (6)	0.411 (11)
H14D	0.5301	0.6342	1.1039	0.199*	0.411 (11)
H14E	0.4770	0.7373	1.0812	0.199*	0.411 (11)
H14F	0.4829	0.6801	1.0175	0.199*	0.411 (11)
C15	0.1684 (4)	0.8095 (3)	0.8315 (2)	0.0765 (11)	
H15A	0.2604	0.8322	0.8200	0.092*	
H15B	0.1699	0.7474	0.8247	0.092*	
C16	0.0772 (5)	0.8692 (4)	0.7713 (2)	0.0898 (13)	
H16A	0.0837	0.9331	0.7727	0.108*	
H16B	−0.0166	0.8512	0.7849	0.108*	
C17	0.1165 (5)	0.8606 (4)	0.6879 (2)	0.0952 (15)	
H17A	0.2069	0.8846	0.6723	0.114*	
H17B	0.1194	0.7959	0.6879	0.114*	
C18	0.0166 (6)	0.9124 (5)	0.6283 (3)	0.130 (2)	
H18A	0.0422	0.9037	0.5767	0.195*	
H18B	0.0172	0.9771	0.6260	0.195*	
H18C	−0.0733	0.8896	0.6442	0.195*	
C19	0.1267 (4)	0.8979 (3)	0.9364 (3)	0.0771 (11)	
H19A	0.0885	0.8906	0.9902	0.093*	
H19B	0.0650	0.9387	0.9006	0.093*	
C20	0.2627 (5)	0.9453 (3)	0.9295 (3)	0.1104 (18)	
H20A	0.3012	0.9576	0.8754	0.133*	
H20B	0.3270	0.9067	0.9650	0.133*	
C21	0.2321 (7)	1.0370 (3)	0.9532 (4)	0.144 (2)	
H21A	0.1911	1.0807	0.9086	0.173*	
H21B	0.1646	1.0253	0.9972	0.173*	
C22	0.3502 (7)	1.0818 (5)	0.9768 (5)	0.164 (3)	
H22A	0.3189	1.1364	0.9921	0.246*	
H22B	0.4154	1.0982	0.9326	0.246*	
H22C	0.3925	1.0396	1.0209	0.246*	
C23	−0.0140 (4)	0.7709 (3)	0.9402 (2)	0.0715 (11)	
H23A	−0.0360	0.7720	0.9949	0.086*	
H23B	−0.0763	0.8141	0.9069	0.086*	
C24	−0.0399 (5)	0.6756 (3)	0.9323 (3)	0.0910 (13)	
H24A	0.0177	0.6302	0.9671	0.109*	
H24B	−0.0201	0.6724	0.8778	0.109*	
C25	−0.1950 (5)	0.6563 (4)	0.9565 (3)	0.1045 (16)	
H25A	−0.2175	0.6711	1.0068	0.125*	
H25B	−0.2514	0.6955	0.9164	0.125*	
C26	−0.2239 (7)	0.5585 (4)	0.9642 (4)	0.138 (2)	
H26A	−0.3190	0.5482	0.9780	0.206*	
H26B	−0.1702	0.5199	1.0051	0.206*	
H26C	−0.2013	0.5439	0.9145	0.206*	
C27	0.3573 (4)	0.3164 (3)	0.3344 (2)	0.0645 (10)	
H27A	0.3649	0.2527	0.3315	0.077*	
H27B	0.2766	0.3438	0.3068	0.077*	
C28	0.4794 (5)	0.3661 (3)	0.2909 (2)	0.0745 (11)	
H28A	0.5618	0.3351	0.3140	0.089*	
H28B	0.4772	0.4287	0.2967	0.089*	
C29	0.4815 (6)	0.3686 (3)	0.2038 (2)	0.0891 (14)	
H29A	0.3959	0.3956	0.1821	0.107*	
H29B	0.4886	0.3058	0.1984	0.107*	
C30	0.5983 (6)	0.4233 (4)	0.1554 (3)	0.1052 (17)	
H30A	0.5935	0.4233	0.1005	0.158*	
H30B	0.6836	0.3956	0.1751	0.158*	
H30C	0.5916	0.4858	0.1600	0.158*	
C31	0.4608 (4)	0.2796 (2)	0.4680 (2)	0.0594 (9)	
H31A	0.5369	0.3184	0.4447	0.071*	
H31B	0.4437	0.2856	0.5219	0.071*	
C32	0.5039 (4)	0.1803 (3)	0.4729 (2)	0.0671 (10)	
H32A	0.4328	0.1397	0.5011	0.081*	
H32B	0.5161	0.1719	0.4195	0.081*	
C33	0.6347 (4)	0.1550 (3)	0.5153 (3)	0.0787 (12)	
H33A	0.6239	0.1678	0.5671	0.094*	
H33B	0.7066	0.1934	0.4851	0.094*	
C34	0.6773 (5)	0.0538 (3)	0.5263 (3)	0.1035 (16)	
H34A	0.7608	0.0412	0.5539	0.155*	
H34B	0.6908	0.0410	0.4751	0.155*	
H34C	0.6072	0.0153	0.5569	0.155*	
C35	0.2118 (4)	0.2592 (3)	0.4525 (2)	0.0656 (10)	
H35A	0.1366	0.2846	0.4188	0.079*	
H35B	0.2311	0.1971	0.4474	0.079*	
C36	0.1655 (4)	0.2532 (3)	0.5383 (2)	0.0748 (11)	
H36A	0.2396	0.2284	0.5730	0.090*	
H36B	0.1415	0.3146	0.5440	0.090*	
C37	0.0430 (5)	0.1918 (4)	0.5630 (3)	0.0986 (15)	
H37A	−0.0319	0.2189	0.5297	0.118*	
H37B	0.0659	0.1320	0.5534	0.118*	
C38	−0.0033 (5)	0.1781 (4)	0.6488 (3)	0.1113 (18)	
H38A	−0.0798	0.1380	0.6607	0.167*	
H38B	−0.0297	0.2367	0.6584	0.167*	
H38C	0.0699	0.1507	0.6823	0.167*	
C39	0.3117 (4)	0.4152 (2)	0.4294 (2)	0.0673 (10)	
H39A	0.3000	0.4123	0.4857	0.081*	
H39B	0.3931	0.4500	0.4092	0.081*	
C40	0.1907 (5)	0.4686 (3)	0.3877 (3)	0.0908 (14)	0.796 (12)
H40A	0.1140	0.4280	0.3948	0.109*	0.796 (12)
H40B	0.2134	0.4899	0.3311	0.109*	0.796 (12)
C41	0.1492 (7)	0.5537 (4)	0.4212 (5)	0.109 (3)	0.796 (12)
H41A	0.0721	0.5861	0.3926	0.131*	0.796 (12)
H41B	0.1196	0.5314	0.4768	0.131*	0.796 (12)
C42	0.2576 (7)	0.6183 (4)	0.4149 (5)	0.146 (2)	0.796 (12)
H42A	0.2252	0.6683	0.4366	0.219*	0.796 (12)
H42B	0.2859	0.6422	0.3600	0.219*	0.796 (12)
H42C	0.3336	0.5873	0.4441	0.219*	0.796 (12)
C40'	0.1907 (5)	0.4686 (3)	0.3877 (3)	0.0908 (14)	0.204 (12)
H40C	0.1125	0.4603	0.4251	0.109*	0.204 (12)
H40D	0.1693	0.4426	0.3445	0.109*	0.204 (12)
C41'	0.213 (3)	0.5728 (7)	0.3538 (9)	0.091 (9)	0.204 (12)
H41C	0.2811	0.5819	0.3103	0.110*	0.204 (12)
H41D	0.1283	0.6024	0.3324	0.110*	0.204 (12)
C42'	0.2576 (7)	0.6183 (4)	0.4149 (5)	0.146 (2)	0.204 (12)
H42D	0.2677	0.6832	0.3912	0.219*	0.204 (12)
H42E	0.3434	0.5913	0.4346	0.219*	0.204 (12)
H42F	0.1905	0.6095	0.4583	0.219*	0.204 (12)
Cd1	0.39579 (3)	0.238778 (19)	0.740476 (17)	0.06795 (13)	
N1	0.1294 (3)	0.8046 (2)	0.91830 (17)	0.0630 (8)	
N2	0.3363 (3)	0.31687 (19)	0.42099 (16)	0.0562 (7)	
O1	0.8225 (3)	−0.0030 (2)	0.8281 (2)	0.0990 (10)	
O2	0.7269 (4)	−0.1637 (2)	0.7821 (3)	0.1111 (12)	
O3	0.4656 (3)	−0.11311 (19)	0.7096 (2)	0.0877 (9)	
O4	0.2312 (4)	0.5948 (2)	0.6119 (2)	0.1084 (11)	
O5	0.0145 (4)	0.6179 (2)	0.7289 (2)	0.1106 (11)	
O6	0.0088 (4)	0.4455 (3)	0.8549 (2)	0.1246 (14)	
S1	0.35964 (11)	0.09033 (7)	0.70076 (7)	0.0724 (3)	
S2	0.62646 (11)	0.17189 (7)	0.78798 (7)	0.0771 (3)	
S3	0.20701 (14)	0.28767 (7)	0.82643 (7)	0.0834 (3)	
S4	0.38097 (11)	0.40010 (7)	0.64893 (6)	0.0682 (3)	
O7	0.9700 (12)	0.1218 (8)	0.8742 (7)	0.086 (3)	0.25
H1O7	0.9367	0.1126	0.8330	0.128*	0.25
H2O7	1.0153	0.1710	0.8597	0.128*	0.25

### Atomic displacement parameters (Å^2^)

**Table:**

	*U*^11^	*U*^22^	*U*^33^	*U*^12^	*U*^13^	*U*^23^
C1	0.0465 (19)	0.0484 (19)	0.0585 (19)	0.0012 (15)	0.0009 (15)	−0.0173 (15)
C2	0.052 (2)	0.054 (2)	0.058 (2)	0.0052 (17)	−0.0039 (16)	−0.0180 (16)
C3	0.052 (2)	0.063 (2)	0.086 (3)	0.0056 (19)	−0.005 (2)	−0.020 (2)
C4	0.057 (2)	0.048 (2)	0.094 (3)	0.0105 (18)	−0.002 (2)	−0.0159 (19)
C5	0.060 (2)	0.051 (2)	0.073 (2)	0.0023 (18)	0.0017 (18)	−0.0209 (18)
C6	0.059 (2)	0.052 (2)	0.061 (2)	−0.0002 (17)	−0.0034 (17)	−0.0198 (17)
C7	0.060 (2)	0.050 (2)	0.054 (2)	−0.0004 (17)	−0.0085 (16)	−0.0176 (16)
C8	0.081 (3)	0.054 (2)	0.075 (3)	−0.004 (2)	−0.019 (2)	−0.013 (2)
C9	0.073 (3)	0.057 (2)	0.094 (3)	0.013 (2)	−0.017 (2)	−0.031 (2)
C10	0.067 (3)	0.068 (3)	0.087 (3)	0.008 (2)	0.004 (2)	−0.029 (2)
C11	0.069 (3)	0.066 (2)	0.084 (3)	0.015 (2)	−0.020 (2)	−0.020 (2)
C12	0.099 (4)	0.072 (3)	0.079 (3)	0.001 (3)	−0.028 (3)	−0.001 (2)
C13	0.132 (13)	0.042 (6)	0.102 (9)	0.000 (7)	−0.036 (8)	−0.023 (5)
C14	0.166 (9)	0.072 (5)	0.095 (7)	0.012 (5)	−0.066 (6)	−0.010 (4)
C12'	0.099 (4)	0.072 (3)	0.079 (3)	0.001 (3)	−0.028 (3)	−0.001 (2)
C13'	0.114 (13)	0.055 (11)	0.111 (14)	−0.006 (10)	−0.054 (10)	0.012 (8)
C14'	0.112 (11)	0.130 (12)	0.166 (13)	0.038 (9)	−0.047 (10)	−0.053 (10)
C15	0.069 (3)	0.089 (3)	0.069 (3)	0.011 (2)	−0.004 (2)	−0.017 (2)
C16	0.084 (3)	0.102 (4)	0.076 (3)	0.016 (3)	−0.013 (2)	−0.010 (2)
C17	0.098 (4)	0.116 (4)	0.068 (3)	−0.003 (3)	−0.009 (2)	−0.017 (3)
C18	0.142 (6)	0.160 (6)	0.083 (3)	−0.009 (5)	−0.032 (4)	−0.016 (4)
C19	0.087 (3)	0.062 (3)	0.079 (3)	0.014 (2)	−0.018 (2)	−0.011 (2)
C20	0.118 (4)	0.062 (3)	0.139 (5)	−0.003 (3)	−0.039 (4)	0.002 (3)
C21	0.143 (5)	0.068 (3)	0.210 (6)	0.001 (3)	−0.044 (5)	−0.008 (4)
C22	0.184 (7)	0.128 (6)	0.191 (7)	0.034 (5)	−0.057 (6)	−0.058 (5)
C23	0.063 (2)	0.080 (3)	0.068 (2)	0.006 (2)	−0.0034 (19)	−0.012 (2)
C24	0.082 (3)	0.088 (3)	0.100 (3)	−0.005 (3)	−0.013 (3)	−0.015 (3)
C25	0.109 (4)	0.103 (4)	0.101 (4)	−0.004 (3)	−0.015 (3)	−0.023 (3)
C26	0.134 (6)	0.118 (5)	0.156 (6)	−0.018 (4)	−0.019 (4)	−0.021 (4)
C27	0.081 (3)	0.055 (2)	0.061 (2)	−0.001 (2)	−0.0143 (19)	−0.0174 (17)
C28	0.095 (3)	0.065 (3)	0.065 (2)	−0.006 (2)	−0.008 (2)	−0.019 (2)
C29	0.120 (4)	0.081 (3)	0.068 (3)	−0.008 (3)	−0.007 (3)	−0.020 (2)
C30	0.138 (5)	0.094 (4)	0.082 (3)	−0.002 (3)	0.013 (3)	−0.024 (3)
C31	0.064 (2)	0.056 (2)	0.059 (2)	−0.0021 (18)	−0.0103 (17)	−0.0147 (17)
C32	0.074 (3)	0.056 (2)	0.071 (2)	0.003 (2)	−0.010 (2)	−0.0138 (18)
C33	0.062 (3)	0.070 (3)	0.097 (3)	0.007 (2)	−0.010 (2)	−0.008 (2)
C34	0.091 (4)	0.084 (3)	0.122 (4)	0.025 (3)	−0.005 (3)	−0.008 (3)
C35	0.065 (2)	0.056 (2)	0.077 (3)	−0.0050 (19)	−0.0149 (19)	−0.0164 (19)
C36	0.070 (3)	0.076 (3)	0.077 (3)	0.001 (2)	−0.009 (2)	−0.016 (2)
C37	0.074 (3)	0.103 (4)	0.117 (4)	−0.012 (3)	0.006 (3)	−0.026 (3)
C38	0.085 (4)	0.127 (5)	0.106 (4)	0.004 (3)	0.015 (3)	−0.007 (3)
C39	0.083 (3)	0.047 (2)	0.078 (2)	0.000 (2)	−0.014 (2)	−0.0242 (18)
C40	0.106 (4)	0.062 (3)	0.112 (4)	0.021 (3)	−0.038 (3)	−0.031 (2)
C41	0.105 (5)	0.066 (4)	0.159 (8)	0.018 (3)	−0.028 (5)	−0.035 (4)
C42	0.136 (6)	0.108 (5)	0.197 (7)	0.012 (4)	−0.011 (5)	−0.046 (5)
C40'	0.106 (4)	0.062 (3)	0.112 (4)	0.021 (3)	−0.038 (3)	−0.031 (2)
C41'	0.095 (19)	0.083 (17)	0.11 (2)	0.002 (14)	−0.006 (15)	−0.047 (15)
C42'	0.136 (6)	0.108 (5)	0.197 (7)	0.012 (4)	−0.011 (5)	−0.046 (5)
Cd1	0.0753 (2)	0.05395 (19)	0.0784 (2)	0.01462 (14)	−0.00680 (15)	−0.02569 (14)
N1	0.0611 (19)	0.065 (2)	0.0605 (18)	0.0078 (16)	−0.0078 (14)	−0.0123 (15)
N2	0.0654 (19)	0.0466 (16)	0.0599 (17)	0.0005 (14)	−0.0146 (14)	−0.0176 (13)
O1	0.067 (2)	0.091 (2)	0.150 (3)	0.0213 (17)	−0.0410 (19)	−0.047 (2)
O2	0.090 (2)	0.0582 (19)	0.186 (4)	0.0213 (17)	−0.031 (2)	−0.030 (2)
O3	0.078 (2)	0.0613 (17)	0.138 (3)	0.0057 (15)	−0.0235 (18)	−0.0479 (18)
O4	0.142 (3)	0.068 (2)	0.098 (2)	0.007 (2)	0.000 (2)	0.0061 (18)
O5	0.115 (3)	0.075 (2)	0.148 (3)	0.037 (2)	−0.024 (2)	−0.041 (2)
O6	0.124 (3)	0.103 (3)	0.136 (3)	0.025 (2)	0.056 (3)	−0.028 (2)
S1	0.0659 (6)	0.0606 (6)	0.0998 (7)	0.0167 (5)	−0.0315 (5)	−0.0341 (5)
S2	0.0676 (7)	0.0607 (6)	0.1173 (9)	0.0053 (5)	−0.0214 (6)	−0.0461 (6)
S3	0.1071 (9)	0.0572 (6)	0.0749 (7)	0.0133 (6)	0.0228 (6)	−0.0051 (5)
S4	0.0782 (7)	0.0620 (6)	0.0644 (6)	−0.0001 (5)	0.0105 (5)	−0.0193 (5)
O7	0.080 (8)	0.082 (8)	0.097 (8)	−0.018 (7)	−0.012 (6)	−0.023 (7)

### Geometric parameters (Å, °)

**Table:**

C1—C2	1.415 (5)	C24—C25	1.577 (6)
C1—C5	1.463 (5)	C24—H24A	0.9700
C1—S1	1.685 (4)	C24—H24B	0.9700
C2—C3	1.451 (5)	C25—C26	1.475 (6)
C2—S2	1.699 (4)	C25—H25A	0.9700
C3—O1	1.224 (5)	C25—H25B	0.9700
C3—C4	1.504 (6)	C26—H26A	0.9600
C4—O2	1.214 (4)	C26—H26B	0.9600
C4—C5	1.490 (5)	C26—H26C	0.9600
C5—O3	1.222 (4)	C27—C28	1.498 (6)
C6—C7	1.402 (5)	C27—N2	1.512 (4)
C6—C10	1.468 (5)	C27—H27A	0.9700
C6—S3	1.683 (4)	C27—H27B	0.9700
C7—C8	1.478 (5)	C28—C29	1.506 (5)
C7—S4	1.693 (4)	C28—H28A	0.9700
C8—O4	1.215 (5)	C28—H28B	0.9700
C8—C9	1.480 (6)	C29—C30	1.520 (6)
C9—O5	1.222 (5)	C29—H29A	0.9700
C9—C10	1.505 (6)	C29—H29B	0.9700
C10—O6	1.209 (5)	C30—H30A	0.9600
C11—C12	1.513 (5)	C30—H30B	0.9600
C11—N1	1.514 (5)	C30—H30C	0.9600
C11—H11A	0.9700	C31—C32	1.513 (5)
C11—H11B	0.9700	C31—N2	1.514 (5)
C12—C13	1.554 (8)	C31—H31A	0.9700
C12—H12A	0.9700	C31—H31B	0.9700
C12—H12B	0.9700	C32—C33	1.501 (5)
C13—C14	1.526 (9)	C32—H32A	0.9700
C13—H13A	0.9700	C32—H32B	0.9700
C13—H13B	0.9700	C33—C34	1.524 (6)
C14—H14A	0.9600	C33—H33A	0.9700
C14—H14B	0.9600	C33—H33B	0.9700
C14—H14C	0.9600	C34—H34A	0.9600
C13'—C14'	1.538 (10)	C34—H34B	0.9600
C13'—H13C	0.9700	C34—H34C	0.9600
C13'—H13D	0.9700	C35—C36	1.518 (5)
C14'—H14D	0.9600	C35—N2	1.519 (5)
C14'—H14E	0.9600	C35—H35A	0.9700
C14'—H14F	0.9600	C35—H35B	0.9700
C15—C16	1.499 (5)	C36—C37	1.518 (6)
C15—N1	1.522 (5)	C36—H36A	0.9700
C15—H15A	0.9700	C36—H36B	0.9700
C15—H15B	0.9700	C37—C38	1.502 (7)
C16—C17	1.520 (5)	C37—H37A	0.9700
C16—H16A	0.9700	C37—H37B	0.9700
C16—H16B	0.9700	C38—H38A	0.9600
C17—C18	1.507 (6)	C38—H38B	0.9600
C17—H17A	0.9700	C38—H38C	0.9600
C17—H17B	0.9700	C39—C40	1.514 (5)
C18—H18A	0.9600	C39—N2	1.529 (4)
C18—H18B	0.9600	C39—H39A	0.9700
C18—H18C	0.9600	C39—H39B	0.9700
C19—N1	1.511 (5)	C40—C41	1.568 (6)
C19—C20	1.524 (4)	C40—H40A	0.9700
C19—H19A	0.9700	C40—H40B	0.9700
C19—H19B	0.9700	C41—C42	1.450 (7)
C20—C21	1.550 (4)	C41—H41A	0.9700
C20—H20A	0.9700	C41—H41B	0.9700
C20—H20B	0.9700	C42—H42A	0.9600
C21—C22	1.492 (5)	C42—H42B	0.9600
C21—H21A	0.9700	C42—H42C	0.9600
C21—H21B	0.9700	C41'—H41C	0.9700
C22—H22A	0.9600	C41'—H41D	0.9700
C22—H22B	0.9600	Cd1—S3	2.5254 (13)
C22—H22C	0.9600	Cd1—S4	2.5296 (13)
C23—C24	1.503 (5)	Cd1—S2	2.5315 (15)
C23—N1	1.511 (5)	Cd1—S1	2.5413 (12)
C23—H23A	0.9700	O7—H1O7	0.8499
C23—H23B	0.9700	O7—H2O7	0.8500
			
C2—C1—C5	108.9 (3)	H25A—C25—H25B	108.1
C2—C1—S1	128.7 (3)	C25—C26—H26A	109.5
C5—C1—S1	122.4 (3)	C25—C26—H26B	109.5
C1—C2—C3	110.7 (3)	H26A—C26—H26B	109.5
C1—C2—S2	127.9 (3)	C25—C26—H26C	109.5
C3—C2—S2	121.4 (3)	H26A—C26—H26C	109.5
O1—C3—C2	128.6 (4)	H26B—C26—H26C	109.5
O1—C3—C4	125.0 (4)	C28—C27—N2	115.9 (3)
C2—C3—C4	106.4 (3)	C28—C27—H27A	108.3
O2—C4—C5	126.5 (4)	N2—C27—H27A	108.3
O2—C4—C3	126.8 (4)	C28—C27—H27B	108.3
C5—C4—C3	106.7 (3)	N2—C27—H27B	108.3
O3—C5—C1	128.0 (4)	H27A—C27—H27B	107.4
O3—C5—C4	124.7 (3)	C27—C28—C29	110.8 (3)
C1—C5—C4	107.3 (3)	C27—C28—H28A	109.5
C7—C6—C10	110.1 (3)	C29—C28—H28A	109.5
C7—C6—S3	128.4 (3)	C27—C28—H28B	109.5
C10—C6—S3	121.5 (3)	C29—C28—H28B	109.5
C6—C7—C8	109.2 (3)	H28A—C28—H28B	108.1
C6—C7—S4	128.4 (3)	C28—C29—C30	113.3 (4)
C8—C7—S4	122.4 (3)	C28—C29—H29A	108.9
O4—C8—C7	126.6 (4)	C30—C29—H29A	108.9
O4—C8—C9	125.9 (4)	C28—C29—H29B	108.9
C7—C8—C9	107.4 (3)	C30—C29—H29B	108.9
O5—C9—C8	127.6 (4)	H29A—C29—H29B	107.7
O5—C9—C10	125.7 (4)	C29—C30—H30A	109.5
C8—C9—C10	106.6 (3)	C29—C30—H30B	109.5
O6—C10—C6	127.9 (4)	H30A—C30—H30B	109.5
O6—C10—C9	125.5 (4)	C29—C30—H30C	109.5
C6—C10—C9	106.6 (3)	H30A—C30—H30C	109.5
C12—C11—N1	116.5 (3)	H30B—C30—H30C	109.5
C12—C11—H11A	108.2	C32—C31—N2	116.8 (3)
N1—C11—H11A	108.2	C32—C31—H31A	108.1
C12—C11—H11B	108.2	N2—C31—H31A	108.1
N1—C11—H11B	108.2	C32—C31—H31B	108.1
H11A—C11—H11B	107.3	N2—C31—H31B	108.1
C11—C12—C13	107.9 (5)	H31A—C31—H31B	107.3
C11—C12—H12A	110.1	C33—C32—C31	111.1 (3)
C13—C12—H12A	110.1	C33—C32—H32A	109.4
C11—C12—H12B	110.1	C31—C32—H32A	109.4
C13—C12—H12B	110.1	C33—C32—H32B	109.4
H12A—C12—H12B	108.4	C31—C32—H32B	109.4
C14—C13—C12	110.3 (8)	H32A—C32—H32B	108.0
C14—C13—H13A	109.6	C32—C33—C34	112.9 (4)
C12—C13—H13A	109.6	C32—C33—H33A	109.0
C14—C13—H13B	109.6	C34—C33—H33A	109.0
C12—C13—H13B	109.6	C32—C33—H33B	109.0
H13A—C13—H13B	108.1	C34—C33—H33B	109.0
C13—C14—H14A	109.5	H33A—C33—H33B	107.8
C13—C14—H14B	109.5	C33—C34—H34A	109.5
H14A—C14—H14B	109.5	C33—C34—H34B	109.5
C13—C14—H14C	109.5	H34A—C34—H34B	109.5
H14A—C14—H14C	109.5	C33—C34—H34C	109.5
H14B—C14—H14C	109.5	H34A—C34—H34C	109.5
C14'—C13'—H13C	109.9	H34B—C34—H34C	109.5
C14'—C13'—H13D	109.9	C36—C35—N2	116.0 (3)
H13C—C13'—H13D	108.3	C36—C35—H35A	108.3
C13'—C14'—H14D	109.5	N2—C35—H35A	108.3
C13'—C14'—H14E	109.5	C36—C35—H35B	108.3
H14D—C14'—H14E	109.5	N2—C35—H35B	108.3
C13'—C14'—H14F	109.5	H35A—C35—H35B	107.4
H14D—C14'—H14F	109.5	C37—C36—C35	110.7 (4)
H14E—C14'—H14F	109.5	C37—C36—H36A	109.5
C16—C15—N1	115.8 (3)	C35—C36—H36A	109.5
C16—C15—H15A	108.3	C37—C36—H36B	109.5
N1—C15—H15A	108.3	C35—C36—H36B	109.5
C16—C15—H15B	108.3	H36A—C36—H36B	108.1
N1—C15—H15B	108.3	C38—C37—C36	113.7 (4)
H15A—C15—H15B	107.4	C38—C37—H37A	108.8
C15—C16—C17	111.2 (4)	C36—C37—H37A	108.8
C15—C16—H16A	109.4	C38—C37—H37B	108.8
C17—C16—H16A	109.4	C36—C37—H37B	108.8
C15—C16—H16B	109.4	H37A—C37—H37B	107.7
C17—C16—H16B	109.4	C37—C38—H38A	109.5
H16A—C16—H16B	108.0	C37—C38—H38B	109.5
C18—C17—C16	111.5 (4)	H38A—C38—H38B	109.5
C18—C17—H17A	109.3	C37—C38—H38C	109.5
C16—C17—H17A	109.3	H38A—C38—H38C	109.5
C18—C17—H17B	109.3	H38B—C38—H38C	109.5
C16—C17—H17B	109.3	C40—C39—N2	116.5 (3)
H17A—C17—H17B	108.0	C40—C39—H39A	108.2
C17—C18—H18A	109.5	N2—C39—H39A	108.2
C17—C18—H18B	109.5	C40—C39—H39B	108.2
H18A—C18—H18B	109.5	N2—C39—H39B	108.2
C17—C18—H18C	109.5	H39A—C39—H39B	107.3
H18A—C18—H18C	109.5	C39—C40—C41	111.5 (4)
H18B—C18—H18C	109.5	C39—C40—H40A	109.3
N1—C19—C20	116.7 (3)	C41—C40—H40A	109.3
N1—C19—H19A	108.1	C39—C40—H40B	109.3
C20—C19—H19A	108.1	C41—C40—H40B	109.3
N1—C19—H19B	108.1	H40A—C40—H40B	108.0
C20—C19—H19B	108.1	C42—C41—C40	114.0 (6)
H19A—C19—H19B	107.3	C42—C41—H41A	108.7
C19—C20—C21	106.2 (4)	C40—C41—H41A	108.7
C19—C20—H20A	110.5	C42—C41—H41B	108.7
C21—C20—H20A	110.5	C40—C41—H41B	108.7
C19—C20—H20B	110.5	H41A—C41—H41B	107.6
C21—C20—H20B	110.5	C41—C42—H42A	109.5
H20A—C20—H20B	108.7	C41—C42—H42B	109.5
C22—C21—C20	116.7 (5)	H42A—C42—H42B	109.5
C22—C21—H21A	108.1	C41—C42—H42C	109.5
C20—C21—H21A	108.1	H42A—C42—H42C	109.5
C22—C21—H21B	108.1	H42B—C42—H42C	109.5
C20—C21—H21B	108.1	H41C—C41'—H41D	107.8
H21A—C21—H21B	107.3	S3—Cd1—S4	87.62 (4)
C21—C22—H22A	109.5	S3—Cd1—S2	125.26 (5)
C21—C22—H22B	109.5	S4—Cd1—S2	119.07 (4)
H22A—C22—H22B	109.5	S3—Cd1—S1	116.04 (4)
C21—C22—H22C	109.5	S4—Cd1—S1	125.49 (4)
H22A—C22—H22C	109.5	S2—Cd1—S1	87.59 (3)
H22B—C22—H22C	109.5	C19—N1—C23	104.1 (3)
C24—C23—N1	116.0 (3)	C19—N1—C11	111.6 (3)
C24—C23—H23A	108.3	C23—N1—C11	112.0 (3)
N1—C23—H23A	108.3	C19—N1—C15	113.0 (3)
C24—C23—H23B	108.3	C23—N1—C15	111.3 (3)
N1—C23—H23B	108.3	C11—N1—C15	105.0 (3)
H23A—C23—H23B	107.4	C27—N2—C31	111.9 (3)
C23—C24—C25	107.0 (4)	C27—N2—C35	106.0 (3)
C23—C24—H24A	110.3	C31—N2—C35	112.0 (3)
C25—C24—H24A	110.3	C27—N2—C39	111.0 (3)
C23—C24—H24B	110.3	C31—N2—C39	105.8 (3)
C25—C24—H24B	110.3	C35—N2—C39	110.2 (3)
H24A—C24—H24B	108.6	C1—S1—Cd1	97.67 (12)
C26—C25—C24	110.6 (5)	C2—S2—Cd1	97.87 (13)
C26—C25—H25A	109.5	C6—S3—Cd1	97.98 (13)
C24—C25—H25A	109.5	C7—S4—Cd1	97.59 (12)
C26—C25—H25B	109.5	H1O7—O7—H2O7	107.7
C24—C25—H25B	109.5		

### Hydrogen-bond geometry (Å, °)

**Table:**

*D*—H···*A*	*D*—H	H···*A*	*D*···*A*	*D*—H···*A*
C12—H12A···O6^i^	0.97	2.48	3.412 (6)	161
C14—H14C···S2^ii^	0.97	2.70	3.472 (10)	137
C16—H16B···O2^iii^	0.97	2.54	3.483 (7)	163

Symmetry codes: (i) −*x*, −*y*+1, −*z*+2; (ii) −*x*+1, −*y*+1, −*z*+2; (iii) *x*−1, *y*+1, *z*.
